# A Classification and Prediction Hybrid Model Construction with the IQPSO-SVM Algorithm for Atrial Fibrillation Arrhythmia

**DOI:** 10.3390/s21155222

**Published:** 2021-08-01

**Authors:** Liang-Hung Wang, Ze-Hong Yan, Yi-Ting Yang, Jun-Ying Chen, Tao Yang, I-Chun Kuo, Patricia Angela R. Abu, Pao-Cheng Huang, Chiung-An Chen, Shih-Lun Chen

**Affiliations:** 1 Department of Microelectronics, College of Physics and Information Engineering, Fuzhou University, Fuzhou 350108, China; eetommy@fzu.edu.cn (L.-H.W.); N191120015@fzu.edu.cn (Z.-H.Y.); N181127067@fzu.edu.cn (Y.-T.Y.); N181120016@fzu.edu.cn (J.-Y.C.); yangtao_fzu@fzu.edu.cn (T.Y.); 2 Department of Information Systems and Computer Science, Ateneo de Manila University, Quezon 1108, Philippines; pabu@ateneo.edu; 3 College of Computer and Information Sciences, Fujian Agriculture and Forestry University, Fuzhou 350002, China; 4 Department of Electrical Engineering, Ming Chi University of Technology, New Taipei 243303, Taiwan; 5 Department of Electronic Engineering, Chung Yuan Christian University, Taoyuan 320314, Taiwan; chrischen@cycu.edu.tw

**Keywords:** ECG signal, atrial fibrillation, support vector machine, image-to-data, prediction

## Abstract

Atrial fibrillation (AF) is the most common cardiovascular disease (CVD), and most existing algorithms are usually designed for the diagnosis (i.e., feature classification) or prediction of AF. Artificial intelligence (AI) algorithms integrate the diagnosis of AF electrocardiogram (ECG) and predict the possibility that AF will occur in the future. In this paper, we utilized the MIT-BIH AF Database (AFDB), which is composed of data from normal people and patients with AF and onset characteristics, and the AFPDB database (i.e., PAF Prediction Challenge Database), which consists of data from patients with Paroxysmal AF (PAF; the records contain the ECG preceding an episode of PAF), and subjects who do not have documented AF. We extracted the respective characteristics of the databases and used them in modeling diagnosis and prediction. In the aspect of model construction, we regarded diagnosis and prediction as two classification problems, adopted the traditional support vector machine (SVM) algorithm, and combined them. The improved quantum particle swarm optimization support vector machine (IQPSO-SVM) algorithm was used to speed the training time. During the verification process, the clinical FZU-FPH database created by Fuzhou University and Fujian Provincial Hospital was used for hybrid model testing. The data were obtained from the Holter monitor of the hospital and encrypted. We proposed an algorithm for transforming the PDF ECG waveform images of hospital examination reports into digital data. For the diagnosis model and prediction model trained using the training set of the AFDB and AFPDB databases, the sensitivity, specificity, and accuracy measures were 99.2% and 99.2%, 99.2% and 93.3%, and 91.7% and 92.5% for the test set of the AFDB and AFPDB databases, respectively. Moreover, the sensitivity, specificity, and accuracy were 94.2%, 79.7%, and 87.0%, respectively, when tested using the FZU-FPH database with 138 samples of the ECG composed of two labels. The composite classification and prediction model using a new water-fall ensemble method had a total accuracy of approximately 91% for the test set of the FZU-FPH database with 80 samples with 120 segments of ECG with three labels.

## 1. Introduction

Cardiovascular disease is the leading cause of death worldwide [1], and the mortality rate from this type of disease continues to increase. Atrial fibrillation (AF) is one of the most common cardiovascular diseases. Clinically, AF can be divided into paroxysmal AF (PAF), persistent AF, and permanent AF according to the time of onset [2]. Before onset, PAF has no significant difference in its ECG and normal ECG, and the time of onset is short and is thus constantly misdiagnosed. Therefore, many researchers have explored various algorithms for analyzing AF.

In 1983, Mood et al. [3] used the Markov process model to calculate the average error value of the prediction array for the RR interval and identify the AF signal of the ECG data. In 2006, Petrucci et al. pointed out that when AF occurs, the RR interval in the ECG has asymmetric distribution characteristics [4]. In 2018, Tang et al. [5] proposed a hybrid Taguchi-genetic algorithm that uses the Gaussian function to decompose the P wave in the ECG signal to improve the performance of the disease classifier. Nurmaini et al. [6] combined discrete wavelet transform and one-dimensional CNN, and the accuracy, sensitivity, and specificity of its classifier reached 99.17%, 98.90%, and 99.17%, respectively.

In the prediction problem, the most important part is to find some features that can predict the occurrence of subsequent phenomena. However, no single feature is found in the prediction of AF with high prediction accuracy [7]. The characteristic of P waves is one of the features. In 2000, Andrikopoulos et al. [8] found that the characteristics of P waves in patients with AF were significantly higher than those in the control group. The P wave variability was defined and became a classic parameter for studying AF with a basis on the characteristics of atrial activity. Lepage et al. [9] cut the P waves after the waveform recognition and combined wavelet transform with the Marco Polo model to improve the specificity and sensitivity.

Overall, the algorithms for AF analysis can be divided into traditional machine learning and deep learning methods. Deep learning algorithms can carry out end-to-end learning, and their capacities depend on their structures. In 2016, Abdul-Kadir et al. [10] constructed an AF classification model with a neural network containing three fully interconnected layers where the inputs were extracted ECG features. The accuracy of the model was 95.3% in the MIT-BIH database. In the 2017 PhysioNet/Computing in Cardiology Challenge, Rubin et al. [11] trained a convolutional neural network (CNN) with a Densely Connected block and utilized 8528 ECG records as inputs. It ranked third among 80 teams in the competition, reporting an F1 score of 0.82. Attia et al. [12] developed a CNN AF prediction model with 10 residual blocks. The data were obtained from 180,922 patients with AF for over 23 years. The prediction accuracy of the model reached 83.3% for the test set. Wang [13] proposed an automatic AF detection method based on an 11-layer neural network. The first eight layers were composed of a CNN, and the last three layers were composed of an improved Elman neural network. The data were obtained from NSR, arrhythmia, and AF data sets from MIT-BIH. The accuracy of the model classification was 97.4%.

Deep learning methods exhibit higher accuracy in the classification of heart diseases than traditional machine learning methods [14,15,16,17], but they have the disadvantages of depending on large data sets, which are difficult to obtain in the field of medical data analysis and have poor interpretability. Deep learning methods require digital databases collected by advanced ECG equipment. The currently available ECG network database has a limited amount of data, and ECG data collected by many hospitals for decades cannot be effectively used for artificial intelligence (AI) heart disease diagnosis due to format concern problems. In contrast, the traditional method is not subject to these restrictions, and the performance of the model can also reach a considerable level [18,19,20,21,22,23].

The SVM has been recognized as an efficient tool for classifying data in a hyperplane [24] and is widely used in ECG analysis. Li et al. [25] constructed two special intuitive grid maps and used the RR interval as the main feature for classifying AF and normal ECG with linear SVM. The sensitivity, specificity, and accuracy of the model were 95.3%, 96.3%, and 95.9%, respectively. Czabanski et al. [26] designed an LSVM classifier with sixteen input features extracted from HRV signals, which achieved the training of a few samples with an excellent classification performance; the classifier had an accuracy rate of 98.86%. Nuryani et al. [27] tested different SVM structures, used radial basis function (RBF), and examined two ECG features as SVM inputs (the average of RR intervals and standard deviation of the RR intervals). The final sensitivity, specificity, and accuracy were 95.81%, 98.44%, and 97.50%, respectively. In general, SVM has a better performance compared to other traditional machine learning methods [28,29] and can be generalized well even with small samples [30]. The related works are listed in Table 1.

In this study, the proposed model was trained and tested on two databases, the MIT-BIH AF database (AFDB) [31] and PAF Prediction Challenge Database (AFPDB) [32,33], and we constructed a two-stage classified-prediction model of AF. The FZU-FPH database was established by Fuzhou University laboratory and Fujian Provincial Hospital [34,35], which was used to test the performance of the model for clinical verification. The analysis of AF was regarded as a classification problem, thus selecting the SVM as the classifier. To test the clinical applicability of the model, a method for converting the PDF ECG image of the hospital into digital data is proposed that can expand the database for model training and testing.

Contributions: This research has various contributions in the domain of intelligence analysis of atrial fibrillation.

1. The proposed classification and prediction hybrid model uses a new waterfall ensemble method. The first step executed the heartbeat classification, classifying normal and AF categories, and then PAF prediction was performed in which the hybrid model was more suitable for clinical applications, with improved accuracy, and realized an intelligent analysis of AF.
2. Second, the research focused on classified features and introduced wavelet changes in the features of AF.
3. Third, research was conducted on improved quantum particle swarm optimization SVM (IQPSO-SVM) training acceleration and used cross-validation to compare the improved acceleration algorithm with the traditional Grid-Search SVM optimization method.
4. Lastly, the proposed algorithm was converted the clinical ECG to digital data, which were acquired from the Holter monitor in the hospital; Thus, the data of the AF database can be greatly increased. The model was validated on the FZU-FPH database, which further reflects the clinical generalization ability of the model.

The paper is organized as follows: Section 2 introduces the materials and methods. Section 3 presents the architecture of the model and explains it in detail in terms of pre-processing, feature extraction, model construction, and clinical data set processing method. The performance evaluation results, corresponding comparison with other published studies, and discussion are shown in Section 4 and Section 5. Finally, Section 6 concludes the paper.

## 2. Materials and Methods

The ECG signals of AF were mainly obtained from three databases. The first database was the MIT-BIH AF database (AFDB). This database contains long-term ECG records of 25 patients with AF. Each record has a duration of 10 h and contains two-channel signals, and each record is sampled at a rate of 250 points per second with a 12-bit resolution at ±10 mV. Its label includes the following: AFIB (AF), AFL (atrial flutter), J (AV nodules), and N (all other rhythms). The database contains the AF ECGs of patients with AF, which were different from the normal ECGs of normal people. The database is indicated as an AF disease if it has an AF label in the 5 min ECG signal. The database includes 1380 AF and 1140 N labels. Therefore, the database was used in training the classification model of AF, that is, in determining whether a segment of ECG is an AF ECG.

The second database is the PAF Prediction Challenge Database (AFPDB) from physionet. This dual-channel ECG database, which was extracted from the Holter monitor, was created in the 2001 Computer in Cardiology Challenge. The database was divided into training and test sets. The record set started with the p tag coming from patients who had PAF but did not have episodes at the time of recording. The record set started with the n tag from healthy people who had no record of PAF at any time. Each data segment is a 5 min recoding. Therefore, 50 normal people and 50 PAF patients with their 30 min data have 300 N and 300 PAF labels, respectively. The database was used in distinguishing the normal ECG records of normal people and patients with PAF and training a predictive model of PAF.

The third database is the FZU-FPH database that was jointly established by Fuzhou University laboratory and Fujian Provincial Hospital. The ECG acquisition equipment used was the DMS300-4A with a sampling frequency of 3000, and the multi-lead data were collected from subjects aged 18–70 from Fujian Province with a 4096-Hz Holter monitor. Each record indicates the gender, age, acquisition time, heartbeat label, and diagnosis conclusion of a patient. The database was used for clinical verification of the model.

## 3. Establishment of the Composite Model

**Figure 1:**
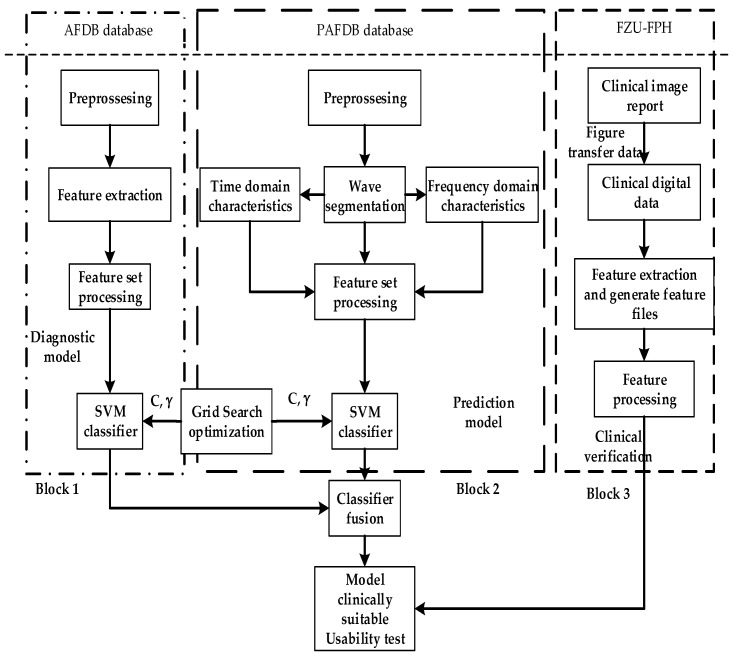
Overall implementation of the ECG analysis algorithm with atrial fibrillation.

**Figure 2:**
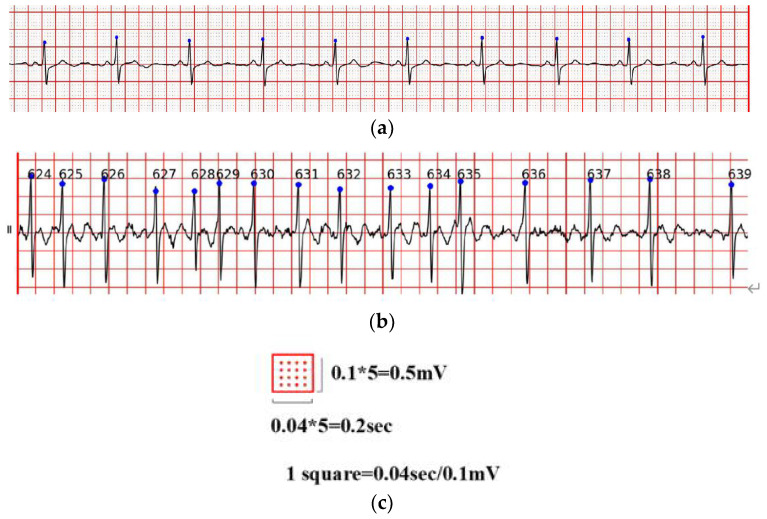
(**a**) Normal ECG, (**b**) atrial fibrillation ECG; (**c**) is the scales of (**a**,**b**).

As shown in Figure 1, the overall ECG analysis algorithm for AF included three blocks: atrial diagnosis model, atrial predict model, and clinical verification part. The first two blocks are composed of three stages: pre-processing, feature extraction, and model training. The data were divided into 5 min sections. In the diagnosis model: after pre-processing, which filters out the interference and R-wave detection, only the time-domain feature was extracted because the difference between normal ECG and AF ECG was significant, as shown in Figure 2a,b, respectively, and their scales is shown in Figure 2c. In the predicted model, given that the normal ECG of patients with AF was similar to normal ECG, wave segmentation to extract features, PR interval, P-wave amplitude, and six frequency-domain features was performed. After the feature set was extracted, normalization, standardization, and regularization were applied. The training set of the AFDB database and AFPDB database was used to train a diagnosis model and prediction model, respectively. Then, the SVM model with an improved quantum–particle–swarm optimization algorithm was adopted. In the clinical verification part, a method for converting clinical images to digital data was used. After the feature set processing, the obtained feature file was used as the clinical verification data of the final model for testing the performance.

### 3.1. Pre-Processing

Two steps were used to pre-process the ECG signal: (1) Eliminating the noise interferences to obtain the clear ECG signal, and (2) locating the R wave, QRS complex, and P wave position to analyze the ECG signal information and obtain the ECG features.

**Figure 3:**
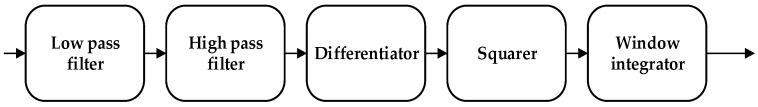
Data pre-processing process.

**Figure 4:**
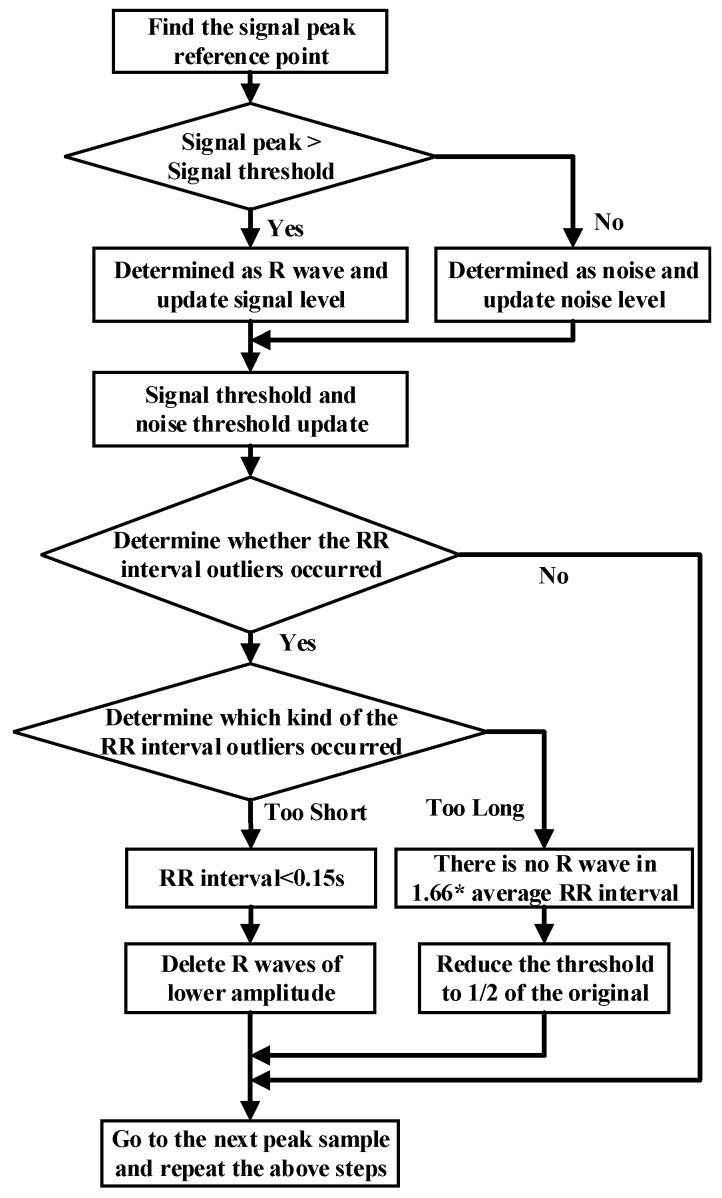
Flow chart of R threshold detection with the dynamic threshold algorithm.

The noise interference of the ECG signal has three parts, namely, power frequency noise, baseline drift, and electromechanical (EMG) interference. The low-pass filter was used to filter the T wave interference, power frequency interference, and higher frequency noise of the ECG signal. The high-pass filter was effectively employed to eliminate the EMG interference and baseline drift. The slope of the R wave was amplified by the proposed differentiator to distinguish the QRS complexes. A nonlinear square function was used to square the signal to enhance the slope of the frequency response and reduce the error caused by the T wave. After squaring, the signal was integrated with a window integrator, thus obtaining the slope information of the R wave and the QRS complex. For the R wave location, the improved Pan–Tompkins [36] algorithm was used to eliminate the misdiagnosis point and compensate for the missed detection point. The pre-processing process diagram and flow-chart of the improved R wave location algorithm are shown in Figure 3 and Figure 4, respectively.

### 3.2. Feature Extraction

Differences were found between the ECG characteristics of AF and normal conditions:

1. The P wave disappeared and was replaced by AF waves with different shapes, spacing, and amplitudes. AF waves were generally obvious in leads V1 and II. The frequency was 350–600 times/min, and the amplitude was generally 0.05–0.50 mV.
2. The QRS complex morphology is usually normal, but when a ventricular bundle branch block or other diseases occur, the QRS complex widens and the morphology appears abnormal.

When AF occurs, the RR interval (RR Interval, RRI) of the patient becomes irregular. By using the irregularity of RRI in extracting relevant parameters, the diagnosis of AF can be improved. In the related research of atrial activity analysis, the relevant characteristics of P waves are mainly used to represent the changes in atrial activity characteristics, such as P wave variability, which is the square of the standard deviation of the P wave interval. However, owing to low amplitude and high frequency and to the replacement of P waves by irregular f waves, which are confused with noise during the detection process, the extraction of P wave intervals is subject to interference. The accuracy of the algorithm for AF detection with atrial activity analysis is not ideal. The data in the AFDB database were collected by Holter limb leads. Compared with the ECG acquired with standard limb leads, one uses the Mason–Likar lead system and the other uses the Einthoven–Wilson lead system. The lead connection position, acquisition equipment parameters, lead wires, and electrodes are different, which causes the P wave shape and ST segment to be different in the ECG signal acquired by the Holter monitor compared with the standard ECG. Therefore, on the basis of the data in the AFDB database as the training set for training the model and evaluating clinical Holter data, features related to the P wave interval were not used for the purpose of offsetting the poor generalization ability of the model in clinical data diagnosis. The relevant features of the RR interval in the ECG were extracted (such as the average value of the RR interval and the standard deviation of the RR interval) for the construction of a classification model of AF, as the ventricular rate was absolutely uneven. In general, the duration of the RR interval in an ECG is stable at about 0.8 s. However, when AF occurs, irregular excitement appears in various parts of the atrial wall. The specific manifestation is that the RR interval changes are unstable and irregular.

After observing all the data in the database, it was noticed that PAF in many cases appeared after premature beats because RRI would change when premature beats occurred. Therefore, in the selection of the first set of features, RR interval-related features were used, which were consistent with the features used in the diagnosis model. In addition to inter-beat characteristics, the prediction model used intra-beat features, such as PR interval and P-wave amplitude, to compare P-wave time domain differences between patients with AF and those with normal sinus rhythm. According to the pathogenesis and pathophysiology of AF, AF is a rapid stimulation of many focal points produced in the atrium. The frequency of these stimuli in the ECG was greater than the P wave frequency and lower than the QRS compound frequency. Therefore, wavelet transformation was performed on the P band of ECG, and the wavelet coefficients generated after transformation were used as the frequency domain features in the selection of the second set of features. The detail coefficient represented the impact of these rapid stimuli on the atrium. The characteristics of the two AF algorithms are shown in Table 2.

In the selection of time domain features, a series of features of ECG based on the inter-beat interval, intra-beat interval, and amplitude features were extracted, and the first set of discriminant features were generated for the prediction of AF.

Interval features between beats: The interval between heartbeats is the interval between two consecutive heartbeats. The peak point of the R wave was used as the reference point in the conducted series of studies on the relevant characteristics of the RR interval. Interval features within beats: Intracardiac interval is the interval between the post-heartbeat reference point and front reference point. The PR interval required for PAF prediction was based on the peak point of the *P* wave and the peak point of the *R* wave, which is the interval between the starting point of the *P* wave and the starting point of the *QRS* complex of a given heartbeat. Its characteristic description is shown in Equation (1), where *i* is the current heartbeat and *on* represents the starting point of the wave.

(1) $$PR=|QRS{\left(i\right)}_{on}-P{\left(i\right)}_{on}|,$$

Amplitude characteristics: The *P* wave amplitude was selected as the PAF prediction feature. Equation (2) is the characteristic description, where *ampP* represents the amplitude of the highest point of P wave amplitude and *ampPon* represents the amplitude of the starting point of *P* wave.

(2) $$ampP_{on}P=\left|ampP-ampP_{on}\right|,$$

In the selection of frequency domain features, the *R* wave peak point detected on the signal sample was used as the reference point, and the interval feature was extracted according to the heartbeat. The feature was based on the regional morphology, and the *P* wave region was extracted using wavelet transform frequency domain characteristics.

A bior5.5 wavelet basis function was used in extracting the P wave features. The biorthogonal (bior Nr. Nd) wavelet is a compactly supported biorthogonal wavelet function, which solves the contradiction between the linear phase and orthogonality requirements. The basic steps of using wavelets for ECG signal processing are as follows:

1. Choose an appropriate wavelet basis function.
2. Decompose the ECG signal into corresponding layers.
3. Keep the coefficients decomposed from a specific layer for the next step of processing.

Given that the frequency span of noise was large, the different dimensions of wavelet transform in processing the signal were used. The process is equivalent to the filtering of signal with different frequencies. Signal characteristics for minimizing the degree of noise interference were obtained.

In general, the wavelet basis function was selected according to the four most obvious characteristics of the wavelet, which are orthogonality, tight support, symmetry, and regularity. Orthogonality is conducive to signal reconstruction. Symmetry reduces the tendency of a signal to be distorted. The tight support length affects the quality of the local characteristics of a signal. The shorter the length, the better the local characteristics. Regularity mainly affects the signal reconstruction smoothing effect. In actual use, the wavelet basis function can be appropriately selected according to the characteristics of the signal and the required results. A high signal-to-noise ratio (SNR) and correlation coefficient of bior5.5 [37] produce the best wavelet basis function used in ECG signal processing.

**Figure 5:**
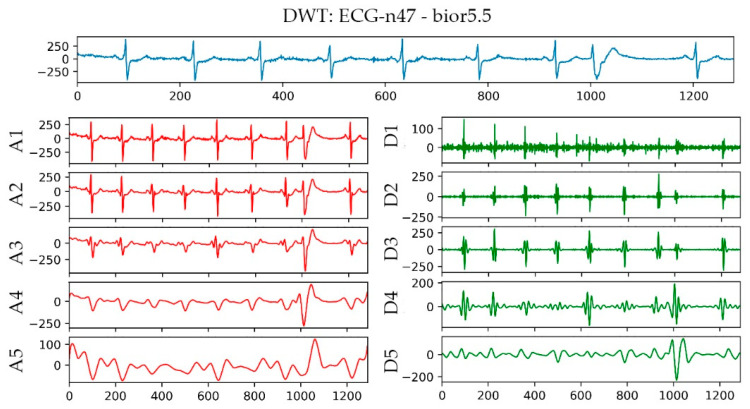
Wavelet transform result graph of No. n47 ECG.

The bior5.5 function was used in performing a 5-layer wavelet transform on the n47 ECG signal in the AFPDB database. The result is shown in Figure 5. The uppermost sub-picture in the figure is the original waveform diagram of ECG No. n47, and A1–A5 are the waveform diagrams reconstructed by the approximation coefficients of different layers of ECG No. n47 after wavelet transformation. D1–D5 are the different layers of ECG No. n47 after wavelet transformation. The waveform image was reconstructed using the detail coefficient. With regard to the detailed parameters of the decomposition of the first and second layers, baseline drift and EMG interference, the *R* wave signal of the third layer and the *P* wave signal of the fourth layer were the most obvious parameters. Therefore, bior5.5 was used as the wavelet basis for wavelet transformation on the P-band, and the approximate coefficients and detail coefficients of the fourth layer were extracted after the transformation as frequency domain features.

The Decision Tree Classification Algorithm was used to evaluate the importance of features. The comparison in Table 3 lists the importance of the features. The amplitude of the P wave was the most relevant feature for patients with atrial fibrillation.

### 3.3. Construction and Verification of the Atrial Fibrillation Model

#### 3.3.1. Construction of the Atrial Fibrillation Model

SVM was used in constructing the AF classifier and PAF predictor. SVM is a two-class classification model and its basis is a linear classifier that maximizes the separation in a feature space for the separation of hyperplanes. The classifier can identify an optimal hyperplane and maximize the interval between two different types of data. In this study, the decision function *f*(*x*) was used to represent the optimal hyperplane learned from the training set and was used in evaluating the classification of the prediction set. The discriminant decision function is expressed as shown in Equation (3):

(3) $$f(x)=sign(\sum_{i=1}^{n}{\vec{\alpha }}_{{}_{i}}^{*}y_{i}K(\vec{x},{\vec{x}}_{i})+b^{*}),$$

In the above equation, ${\vec{\alpha }}^{*}$ is the Lagrangian multiplier that satisfies the optimal solution of the SVM classification problem, and ${\vec{b}}^{*}$ is the shortest distance to the optimal hyperplane. SVM with radial basis function was used as the kernel function in classifying and predicting ECG signals. The discriminant decision function is provided as Equation (4):

(4) $$f(x)=sign(\sum_{i=1}^{n}{\vec{\alpha }}_{{}_{i}}^{*}y_{i}(\exp(-\gamma ||{\vec{x}}_{i}-\vec{x}|{|}^{2}))+b^{*}),$$

When a data set is linearly inseparable, SVM uses the kernel function to calculate and recognize ECG with the different characteristics of the input ECG signal for the classification of AF ECG and sinus ECG and differentiation between the non-onset ECG of PAF and normal ECG.

#### 3.3.2. Improved Quantum–Particle–Swarm–Optimization Support Vector Machine

The Particle-Swarm-Optimization algorithm is essential to swarm intelligence and is mainly used in solving global optimization problems. The algorithm mainly imitates the behavior of bird and fish swarms to enable particles to find global optimal solutions. However, it has two main disadvantages: first, owing to the lack of randomness in the transformation of its particle position, it is easy to fall into the trap of local optimization. Second, it needs to set additional parameters, which will have a certain impact on the optimization of parameters in a composite model. In view of these two shortcomings, an Improved Quantum-Particle-Swarm-Optimization SVM (IQPSO-SVM) was introduced, which can effectively improve the efficiency of SVM parameter optimization without reducing the accuracy of the model. In the classical physical world, the trajectory of a particle can be described by a velocity vector and a position vector. However, in the quantum world, according to the Heisenberg uncertainty principle, the position and velocity of a particle cannot be determined at the same time. If particles during particle swarm optimization have quantum behavior, they will run in different ways. First, the state of a particle is described by the wave function rather than the previous position and velocity. Through the Monte Carlo method, the iterative equation of particle motion can be obtained.

(5) $$\left\{\begin{array}{ll} x_{i}(t+1)=p+\lambda \cdot \left|Mbest_{i}-x_{i}(t)\right|\cdot \ln(1/u), & \;\mathrm{if}\;k⩾0.5\\ x_{i}(t+1)=p-\lambda \cdot \left|Mbest_{i}-x_{i}(t)\right|\cdot \ln(1/u), & \;\mathrm{if}\;k<0.5, \end{array}\right.$$

where λ is the shrinkage–expansion coefficient, also known as the inertia weight or innovation coefficient, u and k are values generated by using the uniform probability distribution function in the range of [0,1], and $M_{best}$ is the average optimal position of all particles, which is obtained by calculating the mean of c with its Equation is as follows:

(6) $$M_{best}=\frac{1}{N}\sum_{i=1}^{N}p_{besti},$$

Inertia weight λ is an important parameter that affects the convergence of IQPSO and is usually considered a fixed value. We improved it in order that λ can change dynamically with the algorithm.

(7) $$\lambda =\lambda_{\max}-\frac{\lambda_{\max}-\lambda_{\min}}{t_{\max}}\times t,$$

where $\lambda_{\max}$ and $\lambda_{\min}$ are the upper and lower bounds of inertia weight and *t* is the number of iterations. In general, the result is better when λ decreases from 1 to 0.5.

**Figure 6:**
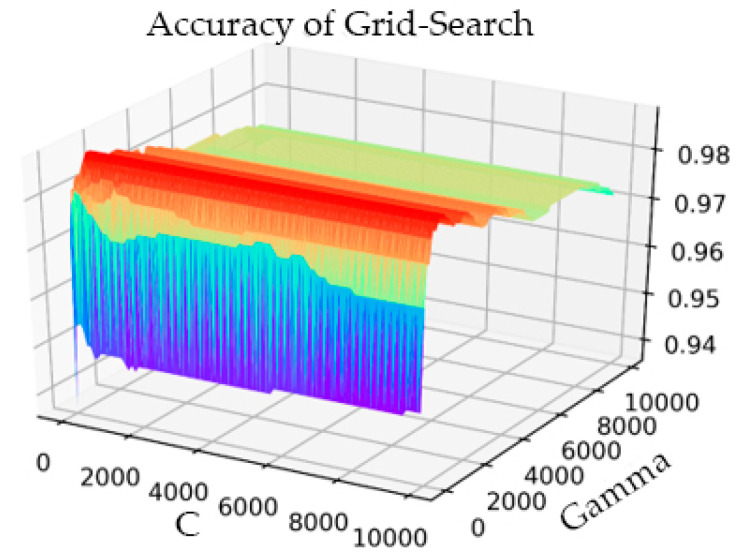
Grid Search parameter optimization process diagram.

In the part of verifying IQPSO, we performed threefold cross-validation to optimize the penalty coefficient C of the SVM and the kernel function parameter gamma with the grid optimization method. The process result is shown in Figure 6.

**Figure 7:**
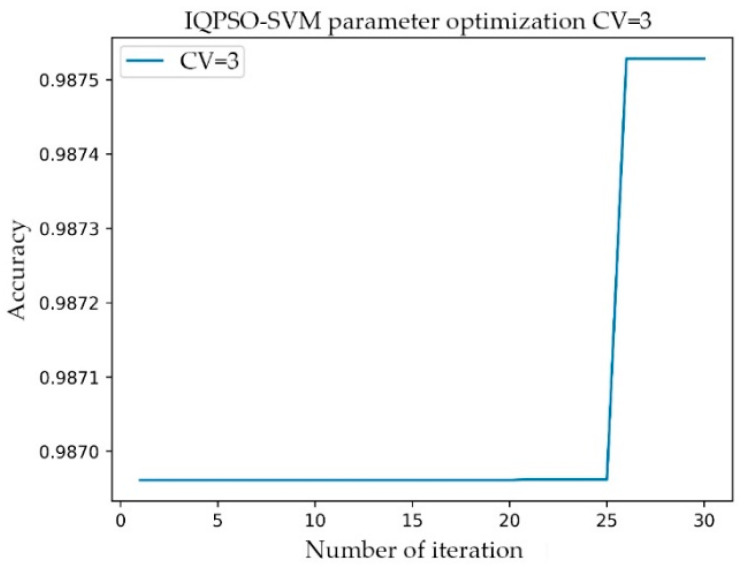
Relationship between the number of iterations and accuracy of IQPSO-SVM.

The setting range of C was 1–10000, the step was 100, and the gamma range was the same as that of C. After the transformation of C and gamma, the accuracy of the model prediction changed. When the parameter C was 101 and gamma was 801, the accuracy rate (Acc) was at its highest, having a value of 0.9873. The simulation time was 1142.914 s. In the training process of the AF classification model, for the same training data, the IQPSO-SVM algorithm designed in this study was used to determine the optimal parameters. Given that the algorithm introduced the average best position $M_{best}$, the ability of the particles to cooperate with each other was enhanced. Moreover, the randomness of the quantum space made it difficult for the algorithm to enter local convergence, and the convergence speed of the model was significantly faster than the existing particle swarm optimization (PSO) and SVM using the grid search. As shown in Figure 7, when the accuracy rate reached 0.9875 after iteration, the recorded processing time was 124.4241 s, which was close to only 1/10 of the processing time of the Grid Search method. The values of the relevant parameters are as follows: C, the range of gamma was 0.001–10000, the number of particle swarms was 20, the number of iterations was 30, and the innovation parameter was 0.6.

Experiments were conducted for the cross-validation of different fold numbers, 3-fold, 5-fold, and 10-fold cross-validation, and comparison of the performance of Grid Search and IQPSO-SVM. The detailed classification accuracy and model tuning time-consuming experimental results are listed in Table 4. The results are represented by the average and deviation. In the case of cross-validation with different fold numbers, the IQPSO algorithm took less time to train a better model. When 10-fold cross-validation was performed, the recorded processing time was 1/20 of the process time of the Grid Search.

#### 3.3.3. Clinical Image Data to Digital Data

**Figure 8:**
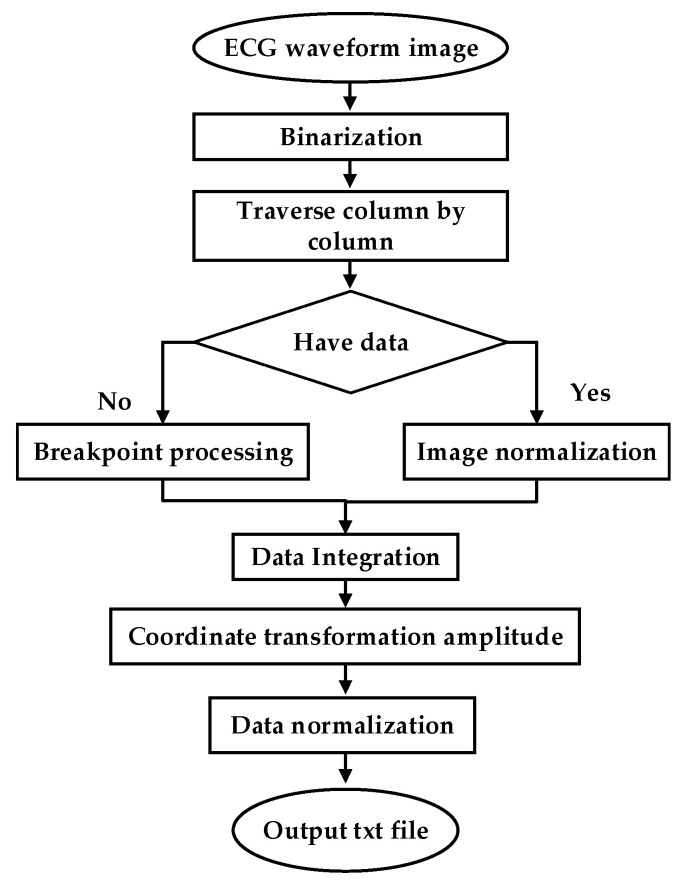
ECG waveform image to data.

To test the clinical applicability of the model, part of the Holter report from the FZU-FPH database was used. The report was a 5 min two-lead image in a 6-page PDF file. Through a series of PDF to data operations, the report was converted into clinical data. The flow chart illustrating the conversion of clinical PDF images to digital data is shown in Figure 8.

**Figure 9:**
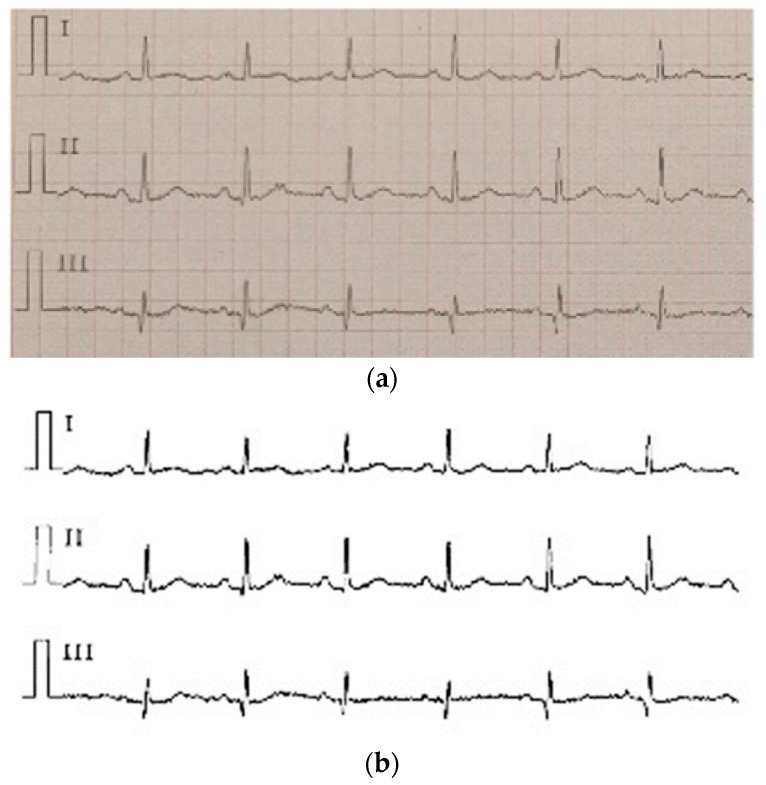
(**a**) the extracted image, (**b**) the original Holter image.

ECG is often difficult to share because of its encrypted feature. Its main form is a paper ECG report, and the main storage format of ECG is a PDF file. In this study, we used the PDF2IMG interface of C++ in image conversion and comprehensively considered the distortion of the ECG image. Finally, the normalized image resolution was determined to be 6614 × 9354; three channels were obtained, and the format used was PNG. For the accurate extraction of the ECG waveform information, background and foreground curves should be separated. This process produces a good digital effect. Based on the OTSU method [38], a process that uses an improved OTSU’s algorithm for separation was proposed. Gamma transformation was performed on the image before the OTSU. This process greatly improved the success rate of curve extraction. In the separation of the curve from the grid, the effective data in this part were eliminated for the generation of breakpoints. This process was necessary because the effective ECG curve and grid intersected. The method involving traversing column by column to add points was adopted. Figure 9a shows the extracted image, and Figure 9b shows the original Holter image.

## 4. Experimental Results and Performance Analysis

The data of the MIT-BIH AF database were used in training the AF classification model. Before training, the experimental data were divided into two groups: a training set and a test set. The training and test sets contained a normal sinus ECG and AF ECG with a balanced amount of data. When SVM is used in training a model, the usual process involves the following procedures: standardization of sample data and samples mapped with a kernel function (the kernel function mostly uses RBF and is linear). When the sample is linearly separable, the linear effect is better than RBF. Given that the research sample in this study was a nonlinear sample, the RBF Gaussian was used as the kernel function, and cross-validation and the Grid Search grid optimization algorithm were used in adjusting the hyperparameters. Finally, the optimal parameters were used in training for the generation of the final model, and the model was tested.

In terms of the performance evaluation of the SVM classification model of AF, accuracy (*ACC*), specificity (*SP*), sensitivity (*SE*), and precision (*P*) were used as the evaluation criteria. The calculation formula is shown in Equations (8)–(10):

(8) $$Acc=\frac{TP+TN}{TP+TN+FP+FN},$$

(9) $$SP=\frac{TN}{TN+FP},$$

(10) SE=Recall(R)=TPTP+FN,

where *TP* represents the proportion of positive samples classified as positive samples, *TN* represents the negative samples classified as negative samples, *FP* represents the proportion of negative samples classified as positive samples, and *FN* is the proportion of positive samples classified as negative samples. Accuracy is the proportion of samples with correct predictions relative to all samples. Specificity represents the proportion of all negative examples that are correctly classified and measures the ability of the classifier to recognize negative examples. Sensitivity represents the proportion of all positive examples that are correctly classified and measures the ability of the classifier to recognize positive examples.

In the AFDB database, the 10 h ECG records of 21 patients were cut down to 5 min records, and 2520 5 min ECG signal segments were obtained. There were 15 patients with 1800 segments in the training set and six patients with 720 segments in the test set. As long as the AF label appeared in the 5 min ECG signal, it was marked as AF disease. The training set included 1020 AF episodes and 780 N episodes. The test set included 360 AF episodes and 360 N episodes. The diagnosis model was trained based on the 1800 segments from the training set and tested with 720 segments from the test set. The classification results obtained are shown in Table 5, in which the x axes and y axes indicate the predicted value and actual value, respectively, and are listed in Table 6, Table 7 and Table 8.

In the AFPDB database, the 30 min data of the normal sinus ECG data of 50 normal people and 50 patients with paroxysmal atrial fibrillation before the onset of the disease were cut down to obtain 600 5 min ECG signal segments. The 5 min ECG signals of normal sinus ECG data were labelled as the N category and others were labelled as PAF. There were 80 people (i.e., 40 normal people and 40 patients) with 480 segments and 20 people (i.e., 10 normal people and 10 patients) with 120 segments that were used as the training set and test set, respectively. Thus, the training set of the prediction model included 240 PAF episodes and 240 N episodes, and the test set included 60 PAF episodes and 60 N episodes. The classification results are shown in Table 6. Since the input signal of the prediction model is a normal ECG signal, the difficulty of its classification is greatly increased. In order to increase the number of test samples and verify the performance of the prediction model, 138 5 min segments from the FZU-FPH database (i.e., 69 ECG signals of normal people and 69 paroxysmal atrial fibrillation patients) were employed to test the performance of the prediction model. The result is listed in Table 7.

**Figure 10:**
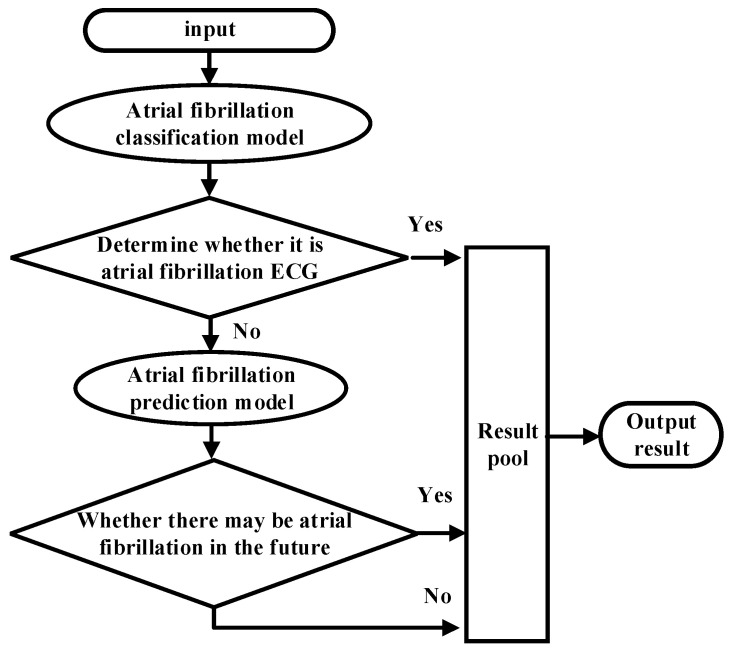
Flow chart of the waterfall fusion algorithm.

For the intelligent analysis of AF by the model, the classification model and predictive model were integrated through the waterfall hybrid method, and these two models were hybridized in a series. The model identification after hybridization is shown in Figure 10. In order to test the performance of the hybrid model, an extra 80 samples with 120 segments were selected from the FZU-FPH, which are different from the 138 samples mentioned above. The ECG signals at the onset of atrial fibrillation, 20 to 30 min before the onset of atrial fibrillation, and normal ECG signals were labelled as AF, PAF, and N diseases, respectively. The hybrid model first classifies whether the signal is the ECG at the onset of atrial fibrillation or not, namely, AF and N categories. In the N category, the predictive model of atrial fibrillation plays a role, identifying whether it is a normal ECG of patients with paroxysmal atrial fibrillation and classifying the N category into N (i.e., normal person) and PAF (i.e., paroxysmal atrial fibrillation patients).

A hybrid model was used to classify and predict the N, AF, and PAF categories after the classification and prediction models were integrated into a hybrid model. The classification results between N and AF categories were significantly different, and the accuracy of the model for N and AF classification was 100%. However, the morphology and features of PAF were between N and AF, and the classification results of PAF were easy to misclassify to the N or AF category. The impact of misclassification of PAF into N was much greater than that of N into PAF. The latter can be cancelled by subsequent medical monitoring. The classification results of fusion models are shown in Table 8, and the performance of different models in different test sets is shown in Table 9.

The calculation methods for the accuracy, sensitivity, and specificity of the three labels are described in Table 9, and the confusion matrix is shown in Table 10. Take N samples as an example, the true positives of N samples are actually the 5 min segments with N labels, and the model recognizes exact N labels, TP_N_ = N_N_. The false positives of the N-type samples indicate that they are not actually the N-type labels, but are predicted to be N-type labels by the model, FP_N_ = PAF_N_ + AF_N_. The true negatives of the N-type samples mean that the actual values are not the N-type labels and the model is not mistakenly classified into the N-type labels, TN_N_ = PAF_PAF_ + PAF_AF_ + AF_PAF_ + AF_AF_. The false negatives of the N-type samples are the actual value of the N-type labels, but they are predicted to be another label, FN_N_ = N_PAF_ + N_AF_. The classification accuracy of N samples is N_ACC_ = (TP_N_ + TN_N_)/(TP_N_ + TN_N_ + FP_N_ + FN_N_). The accuracy of AF labels and PAF labels can be calculated in the same way, and the sensitivity and specificity can be achieved after recognizing the corresponding TP, TN, FP, and FN correctly.

## 5. Discussion

A method for automatically detecting AF episodes was described in this study. The method provides a novel means of extracting features from different databases with SVM. The performance of the proposed method was evaluated using the AFDB, AFPDB, and FZU-FPH databases. Fed with 14 features describing time and frequency domains, the SVM classifier had a sensitivity of 99.2%, specificity of 99.2%, and accuracy of 99.2% for the diagnosis model in the AFDB database test set. A sensitivity of 93.3%, specificity of 91.7%, and accuracy of 92.5% were achieved for the prediction model used in the AFPDB database test set. The particle swarm optimization algorithm considerably improved the simulation time and showed good performance. This research combined the diagnosis and prediction of AF and combined the AI algorithm with AF analysis. The training set and test set of the above-mentioned classification and prediction hybrid model were 5 min ECG signals, which were cut from 30 min ECG signals, and were used to train and test the hybrid model. In order to verify the clinical generalization ability of the model, the FZU-FPH database was used.

Table 11 shows the comparison between the performances of the AF-classified model and the previous research models. As indicated in Table 11, the previous works were tested in the AFDB database except literature sources [8,9]. The proposed method demonstrated excellent discrimination with sensitivity, specificity, and accuracy all exceeding 99% for the AFDB test set. Compared with the performance of the previous traditional algorithm, the performance in the test set of the AFDB database improved. For the use of time-domain-related features, Tateno et al. [37], Li et al. [25], Andersen et al. [18], and Kumar et al. [21] used RRI or some time-domain relative features. The above four literature sources [18,21,25,37] all used the AFDB database as their training material and test set. In 2019, Czabanski et al. [26] used HR features based on RRI and extracted the features of HRV combined with LSVM; the sensitivity, specificity, and accuracy were 98.94%, 98.39%, and 98.66%, respectively. The training heartbeats only played a little part in all heartbeats, but some test set came from the same subject in the training set. For features that use P waves, when deep learning methods are not used, the model performance is greatly reduced. The performance of the model increases with the number of combined features. In [6], the author combined discrete wavelet transform and a one-dimensional convolutional neural network. The training and test material came from four different databases. The classification of the model was excellent but had the disadvantages mentioned before, that is, the amount of data required is relatively large. The classification model proposed in this study can achieve an acceptable accuracy while the training data are insufficient. Through the selection of signal processing algorithms, optimization of a series of algorithms in the processing process and the features selected for research was conducted.

The sensitivity, specificity, and accuracy of the PAF prediction model for the divided test set of the AFPDB database were 93.3%, 91.7%, and 92.5%, respectively. Table 12 shows the comparison between the performance of the PAF prediction model and the previous research model. All studies used AFPDB databases as training and testing material. In [39,40,41], a 30 min ECG signal was used for training and for extracting relevant features. Dozens of features are related to HRV, and different modes of feature training cause differences in model performance. In [42,43], 5 min ECG signals were used for model training. The short ECG sequence contained limited information. The importance of feature selection increases with the number of requirements for the classification performance of a model. In [44,45], both studies extracted HRV relative features as the model input, and showed an excellent performance. However, the number of subjects in their training set and test set was insufficient. A verification step was added in the subjects in the FZU-FPH database, in which the subjects and data of the test set were from 69 ECG signals of normal people and 69 paroxysmal atrial fibrillation patients in hospitals. In [12], the database of Team A was twenty years of data collected with partner hospitals. The amount of data was large, and the overall accuracy rate was 83.3%. The database demonstrated strong clinical generalization capabilities. The sensitivity, specificity, and accuracy in the FZU-FPH database were 94.2%, 79.7%, and 87.0%, which has a better sensitivity compared to literature [12].

To evaluate the clinical generalization ability of the model, we used the model in clinical trials in which the clinical test set data were obtained from the PDF classification reports including 69 segments of PAF and 69 segments of normal control data. The data came from 69 paroxysmal atrial fibrillation and 69 normal subjects in the FZU-FPH database. Each data point had a duration of 5 min. The prediction model had better sensitivity, less misjudgment, and low specificity in predicting PAF, and normal ECG was easily misdiagnosed as sinus ECG of PAF. However, the clinical impact of this misjudgment was smaller than that of sinus ECG in patients with PAF. Therefore, the model has a certain reference and application value in the clinic.

## 6. Conclusions

Despite serious medical consequences, AF is still an underestimated clinical and classification problem. The analysis of AF should be intelligent and reduce the burden on doctors. The normal classification of the ECG of patients with AF is a significant research topic since it can provide timely warning for patients with PAF. In this study, starting from the positioning of the reference point of the ECG II-lead signal, the improved dynamic threshold algorithm was used for the accurate extraction of the R wave. On this basis, the relevant features that include six time-domain features were extracted, as well as the model for the automatic classification model of AF. After a series of investigations on the precursors of PAF, the AF prediction from eight time-domain, and six frequency-domain features, we used an improved quantum particle swarm optimization (IQPSO) algorithm combined with SVM to construct a model for the effective prediction of AF. Finally, the classification and prediction hybrid model was established based on the SVM algorithm using a new waterfall ensemble method. This classifier first diagnosed AF on the input ECG data and then identified AF ECG, and non-AF ECG. The sensitivity, specificity, and accuracy were recorded as 99.2%, 99.2%, and 99.2%, respectively, in the AFDB test set. The model analyzed the factors of primary AF, identified the ECG and normal ECG during the onset period of paroxysmal fibrillation and provided early warning for hidden patients. The sensitivity, specificity, and accuracy were 93.3%, 91.7%, and 92.5%, respectively, in the AFPDB test set and 94.2%, 79.7%, and 87.0%, respectively, in the FZU-FPH clinical test set. The classification accuracy of the hybrid model after the three labels of data were used as an input was approximately 91%. The limitation of this research is focused on the feature of atrial fibrillation without comparison with other classifiers. Future improvements include an end-to-end approach and reducing the manual intervention.

## Figures and Tables

**Table 1:** Summary of related work in recent years.

Works	Year	Model	Features	Methods
Abdul-Kadir et al. [10]	2016	ANN	ECG features	Deep learning method
Rubin et al. [11]	2018	CNN	ECG records	Deep learning method
Attia et al. [12]	2019	CNN	ECG records	Deep learning method
Wang [13]	2020	CNN/ENN	ECG records	Deep learning method
Cai et al. [14]	2020	DDNN	ECG records	Deep learning method
Cao et al. [15]	2020	ResNet	ECG records	Deep learning method
Li et al. [25]	2017	SVM	RR interval	Traditional machine learning method
Czabanski et al. [26]	2020	LSVM	HRV	Traditional machine learning method
Nuryani et al. [27]	2015	SVM	RR interval	Traditional machine learning method
Nuryani et al. [29]	2017	SVM	RR interval	Traditional machine learning method

**Table 2:** Predicted characteristics of atrial fibrillation.

Algorithm Class	Feature Group	Characterization
Atrial fibrillation diagnosis algorithm	Time domain characteristics	RR_std, RR_mean, RR_max, RR_rms RR_cha, RR_len
Atrial fibrillation prediction algorithm	Time domain characteristics	RR_std, RR_mean, RR_max, RR_rms, RR_cha, RR_len, PR, Pamp
	Frequency domain characteristics	ca1,ca2,ca3 cd1,cd2,cd3

**Table 3:** Importance of selected features.

Features	Impact
Pamp	0.268804
cd3	0.220801
RR_std	0.107348
ca3	0.087449
RR_cha	0.081577
PR	0.065737
RR_max	0.058773
ca2	0.027738
cd1	0.021885
RR_rms	0.018379
cd2	0.018140
RR_len	0.012802
ca1	0.010556

**Table 4:** Grid Search and IQPSO-SVM performance comparison.

Cross-Validation	Grid Search		IQPSO-SVM	
	Accuracy	Time(s)	Accuracy	Time(s)
3-fold	0.9836 ± 0.0037	1137.865 ± 5.312	0.9860 ± 0.0018	125.126 ± 1.012
5-fold	0.9810 ± 0.0011	1558.365 ± 6.352	0.9847 ± 0.0009	156.344 ± 2.895
10-fold	0.9835 ± 0.0033	7550.963 ± 5.813	0.9879 ± 0.0010	363.591 ± 3.552

**Table 5:** Classification results of the diagnosis model in the AFDB test set.

	AF	N
**AF**	357	3
**N**	3	357

**Table 6:** Classification results of the prediction model in the AFPDB test set.

	PAF	N
**PAF**	57	3
**N**	4	56

**Table 7:** Classification results of the prediction models in the FZU-FPH test set.

	PAF	N
**PAF**	65	4
**N**	8	61

**Table 8:** Classification results of the fusion model in the FZU-FPH test set.

	N	PAF	AF
**N**	35	5	0
**PAF**	2	37	1
**AF**	0	1	39

**Table 9:** Performance of different models in different test sets.

Performance of Atrial Fibrillation Classification Models in the AFDB Test Set.				
		SE	SP	ACC
AFDB Test Set	AF	99.2%	99.2%	99.2%
AFPDB Test Set	PAF	93.3%	91.7%	92.5%
FZU-FPH Clinical Test Set of Two Labels	PAF	94.2%	79.7%	87.0%
FZU-FPH Clinical Test Set of Three Labels	PAF	92.5%	90%	90.8%
	AF	97.5%	98.8%	98.3%

**Table 10:** Confusion matrix-related definition.

	N	PAF	AF
**N**	N_N_	N_PAF_	N_AF_
**PAF**	PAF_N_	PAF_PAF_	PAF_AF_
**AF**	AF_N_	AF_PAF_	AF_AF_

**Table 11:** Performance comparison of atrial fibrillation classification models.

Works	Characteristic	Methods/Database	SE	SP	ACC
This work	RRI	SVM/AFDB	99.2%	99.2%	99.2%
Tateno et al. [37]	RRI	Coefficient of Variation/AFDB	94.4%	97.2%	------
Li et al. [25]	RRI	LSVM/AFDB	95.9%	95.3%	96.3%
Kumar et al. [21]	ECG features	Random forest/AFDB	95.8%	97.8%	96.8%
Andersen et al. [18]	RRI ECG features	SVM/AFDB	96.81%	96.20%	96.45%
Czabanski et al. [26]	HR features	LSVM/AFDB	98.94%	98.39%	98.66%
Andrikopoulos et al. [8]	P wave	Statistical methods/Self-built database	88%	75%	------
Lepage et al. [9]	P wave	Markov Models/Self-built database	70%	65%	------
Nurmaini et al. [6]	RRI P wave	CNN DWT/AFDB	99.91%	99.91%	99.98%

**Table 12:** Performance comparison of predictive models for paroxysmal atrial fibrillation.

Works	Signal Length (min)	Feature/Methods/Database	SE	SP	ACC
This work (AFPDB)	5	P wave RRI/SVM/AFPDB	93.3%	91.7%	92.5%
This work (FZU-FPH)	5	P wave RRI/SVM/FZU-FPH	94.2%	79.7%	87.0%
Costin et al. [40]	30	HRV and PACs /Statistical analysis/AFPDB	89.3%	89.4%	89.4%
Mohebbi et al. [41]	30	HRV/SVM/AFPDB	96.2%	93.1%	94.5%
Boon et al. [39]	30	HRV/SVM/AFPDB	81.1%	79.3%	80.2%
Xin et al. [43]	5	HRV multi-scale wavelet entropy/SVM/AFPDB	92.18%	94.88%	89.48%
Boon et al. [42]	5	HRV/SVM/AFPDB	86.8%	88.7%	87.7%
Elias et al. [44]	5	HRV/ME/AFPDB	100%	95.55%	98.21%
Parsi et al. [45]	5	HRV/SVM/AFPDB	98.8%	96.7%	97.7%
Attia et al. [12]	----	…/CNN/Self-built database	82.3%	83.4%	83.3%
